# Supplementary material for: Investigating the influence of physiologically relevant hydrostatic pressure on CHO cell batch culture
Source: Sci Rep. 2021 Jan 8;11:162. doi: 10.1038/s41598-020-80576-8 (PMC7794228; doi:10.1038/s41598-020-80576-8)
Supplement: Supplementary file 1 — Supplementary Information 1. [file 41598_2020_80576_MOESM1_ESM.zip › S1/IgG/Feb17 60 90 standard/standard/0mmHg 2.pdf]

Data File C:\Users\P...ts\ChemStation\1\Data\TK_CHO_IgG 2019-02-17 17-28-56\008-1-0ug_ml.D
Sample Name: 0ug/ml

===

Acq. Operator : SYSTEM   Seq. Line : 8
Sample Operator : SYSTEM
Acq. Instrument : HPLC-0039   Location : 1
Injection Date : 2/17/2019 6:00:27 PM   Inj : 1
Inj Volume : 100.000 µl
Different Inj Volume from Sample Entry! Actual Inj Volume : 50.000 µl
Method : C:\Users\Public\Documents\ChemStation\1\Data\TK_CHO_IgG 2019-02-17 17-28-56\CHO_IgG_LC.M (Sequence Method)
Last changed : 2/8/2019 3:17:23 PM by SYSTEM

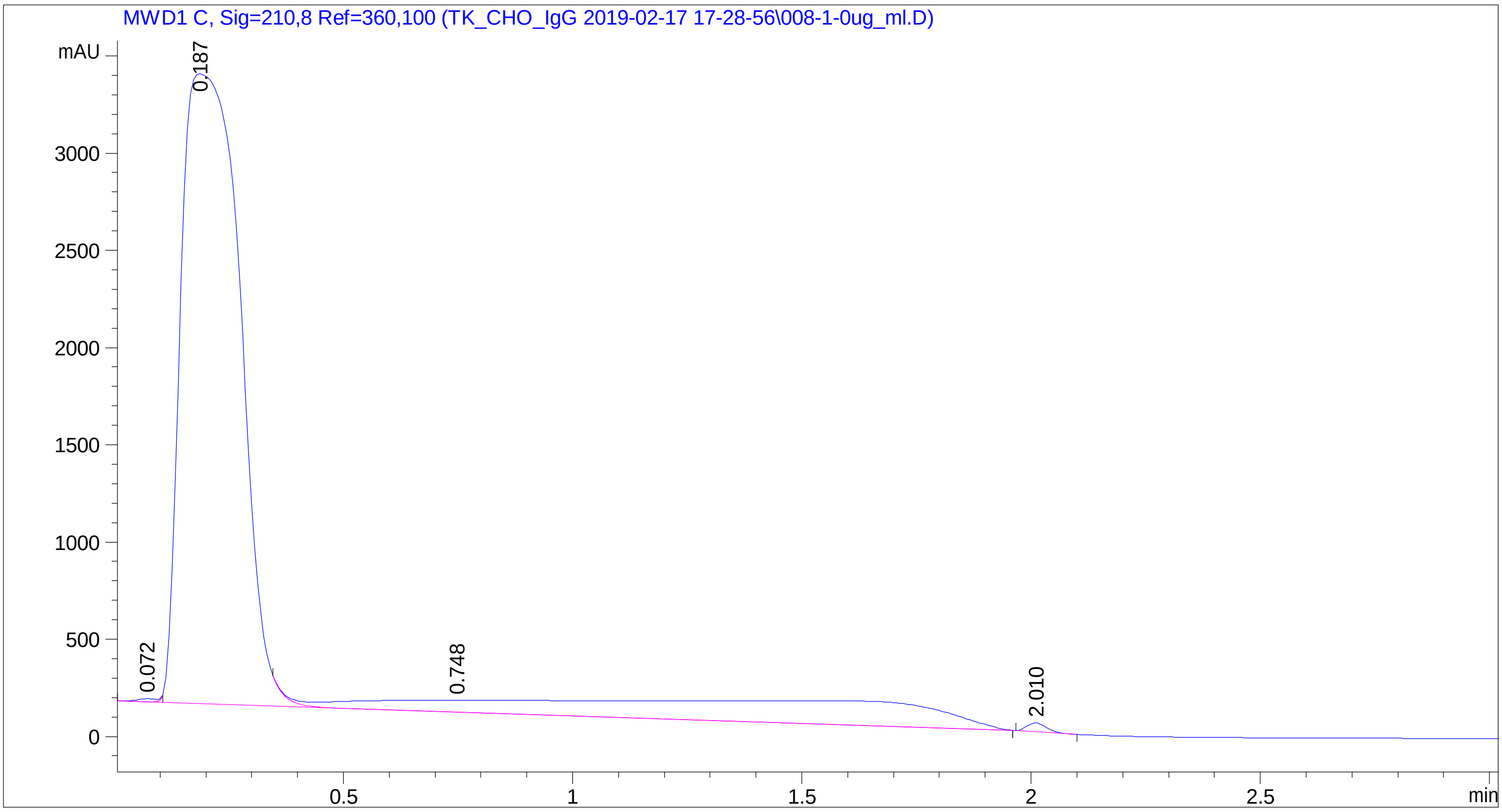

===

Area Percent Report

===

Sorted By : Signal
Multiplier : 1.0000
Dilution : 1.0000
Use Multiplier & Dilution Factor with ISTDs

Signal 1: MWD1 C, Sig=210,8 Ref=360,100

Peak #	RetTime [min]	Type	Width [min]	Area [mAU*s]	Height [mAU]	Area %
1	0.072	BV E	0.0461	47.58399	16.34580	0.1305
2	0.187	VV R	0.1460	2.88698e4	3239.76855	79.1907
3	0.748	VB E	1.4537	7404.40332	60.67829	20.3105
4	2.010	BBA	0.0464	134.24435	45.46957	0.3682

Totals : 3.64560e4 3362.26222

===

*** End of Report ***
